# dl-Piperidinium-2-carboxyl­ate bis­(hydrogen peroxide): unusual hydrogen-bonded peroxide chains

**DOI:** 10.1107/S205698902000972X

**Published:** 2020-07-21

**Authors:** Mger A. Navasardyan, Dmitry A. Grishanov, Petr V. Prikhodchenko, Andrei V. Churakov

**Affiliations:** a Institute of General and Inorganic Chemistry, Russian Academy of Sciences, Leninskii prosp. 31, Moscow 119991, Russian Federation

**Keywords:** peroxosolvates, aliphatic amino acids, charge assisted hydrogen bonds, peroxide H-bonded chains, carboxyl­ate anions, crystal structure

## Abstract

The title compound, C_6_H_11_NO_2_·2H_2_O_2_, is the richest (by molar ratio) in hydrogen peroxide among the peroxosolvates of aliphatic α-amino acids. The asymmetric unit contains a zwitterionic pipecolinic acid mol­ecule and two hydrogen peroxide mol­ecules. The two crystallographically independent hydrogen peroxide mol­ecules form a different number of hydrogen bonds: one forms two as donor and two as acceptor ([2,2] mode) and the other forms two as donor and one as acceptor ([2,1] mode). The latter hydrogen peroxide mol­ecule forms infinite hydrogen-bonded hydro­peroxo chains running along the *c*-axis direction, which is unusual for aliphatic α-amino acid peroxosolvates.

## Abstract 2

The title compound, C_6_H_11_NO_2_·2H_2_O_2_, is the richest (by molar ratio) in hydrogen peroxide among the peroxosolvates of aliphatic α-amino acids. Unusually for aliphatic α-amino acid peroxosolvates, the H_2_O_2_ mol­ecules are linked, forming infinite hydrogen-bonded hydro­peroxo chains running along the *c*-axis direction.

## Chemical context   

Peroxosolvates are crystalline adducts of hydrogen peroxide with various organic or inorganic compounds. Since they are convenient solid sources of active oxygen, some of them have become widely used commercial bleaching, disinfection and oxidation reagents (Jakob *et al.*, 2012 ▸; Cronin *et al.*, 2017 ▸). It is well known that their stability is strongly dependent on the hydrogen-bonded motifs formed by hydrogen peroxide (Chernyshov *et al.*, 2017 ▸). On other hand, H_2_O_2_ is one of the most important signalling mol­ecules in biological systems (Li *et al.*, 2020 ▸; To *et al.*, 2020 ▸). The structures of amino acid peroxosolvates have been studied intensively as simple models of hydrogen peroxide binding with proteins (Prikhodchenko *et al.*, 2011 ▸; Kapustin *et al.*, 2014 ▸). Peroxide and water–peroxide clusters are now of special inter­est since they may simulate cooperative hydrogen-bonded switching in the transportation of hydrogen peroxide species through cell membranes (Grishanov *et al.*, 2017 ▸; Varadaraj & Kumari, 2020 ▸; Wang *et al.*, 2020 ▸). Recently, several structures of organic peroxosolvates with peroxide hydrogen-bonded 1D-aggregates have been reported (Chernyshov *et al.*, 2017 ▸; Navasardyan *et al.*, 2017 ▸, 2018 ▸).
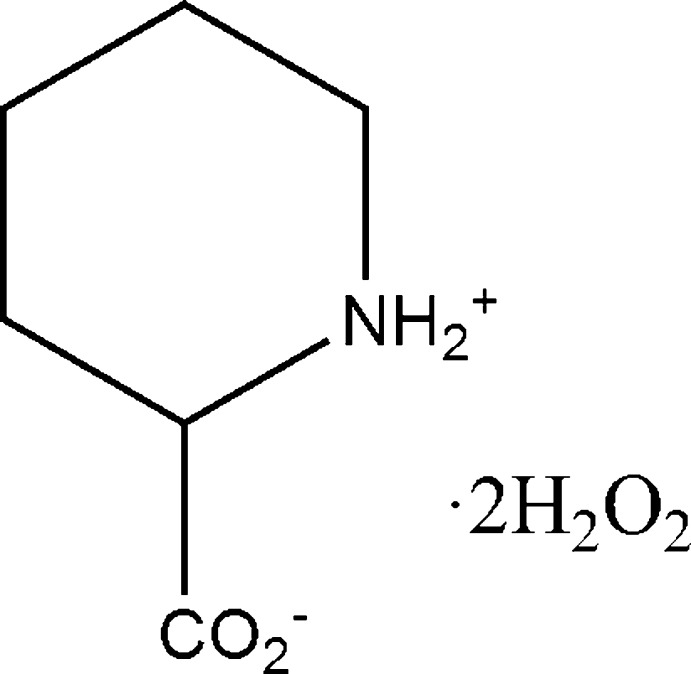


## Structural commentary   

**Figure 1:**
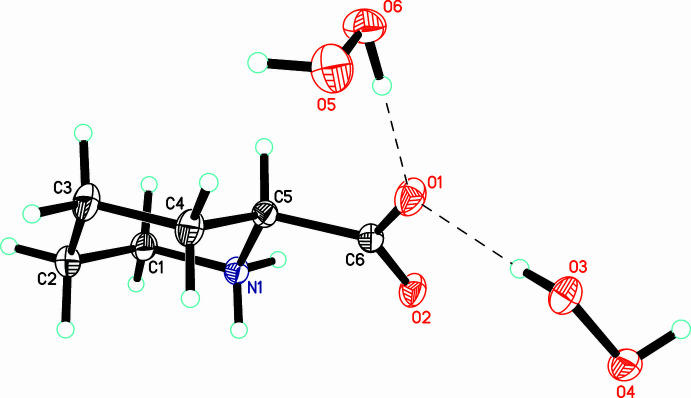
The asymmetric unit of (I) with the atom-numbering scheme. Displacement ellipsoids are drawn at the 50% probability level. Hydrogen bonds are shown as dashed lines.

The asymmetric unit of the title compound (I) comprises a pipecolinic acid mol­ecule and two crystallographically independent peroxide mol­ecules (Fig. 1 ▸). As expected, the amino acid coformer exhibits the zwitterionic form with almost equal C—O distances [1.2429 (11) and 1.2639 (11) Å]. All bond lengths and angles in the organic coformer are close to those observed in the structures of pure pipecolinic acid [(II); Stapleton & Tiekink, 2001 ▸) and pipecolinic acid tetra­hydrate [(III); Bhattacharjee & Chacko, 1979 ▸; Lyssenko *et al.*, 2006 ▸]. As observed for (II) and (III), the pipecolinic acid mol­ecule in (I) adopts a chair conformation with the carboxyl­ate group occupying the equatorial position. It is of inter­est to note in all three structures (I), (II), and (III), the core amino acid fragments N—C—CO_2_ are almost planar, with N—C—C—O torsion angles of less than 22°. This is obviously caused by electrostatic inter­actions between the oppositely charged amino and carb­oxy­lic groups.

## Supra­molecular features   

**Figure 2:**
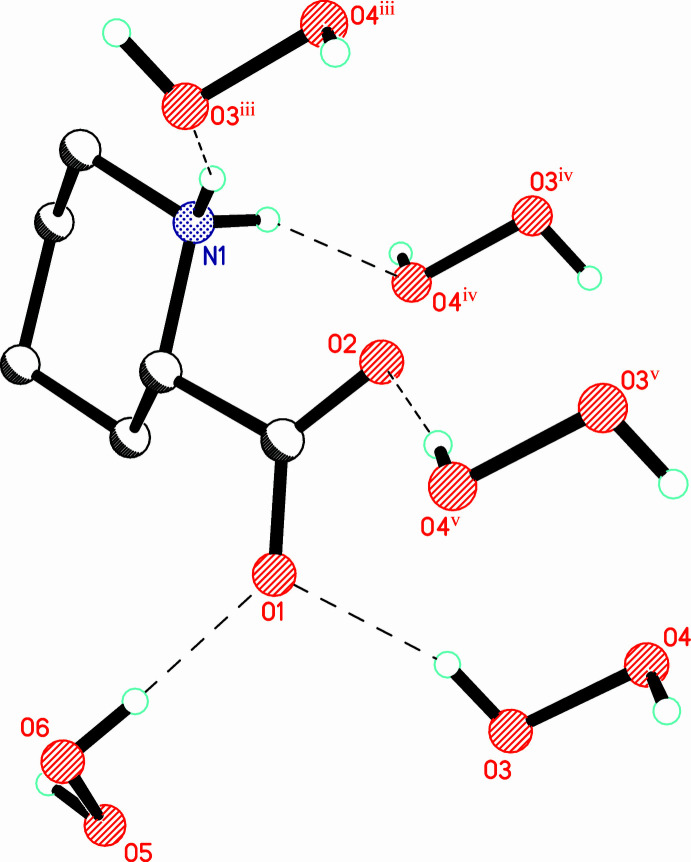
Pipecolinic acid with neighbouring hydrogen-bonded mol­ecules. Hydrogen bonds are shown as dashed lines. [Symmetry codes: (iii) −1 + *x*, *y*, *z*; (iv) 1 − *x*, 1 − *y*, 1 − *z*; (v) 1 − *x*, 1 − *y*, −*z*.]

**Figure 3:**
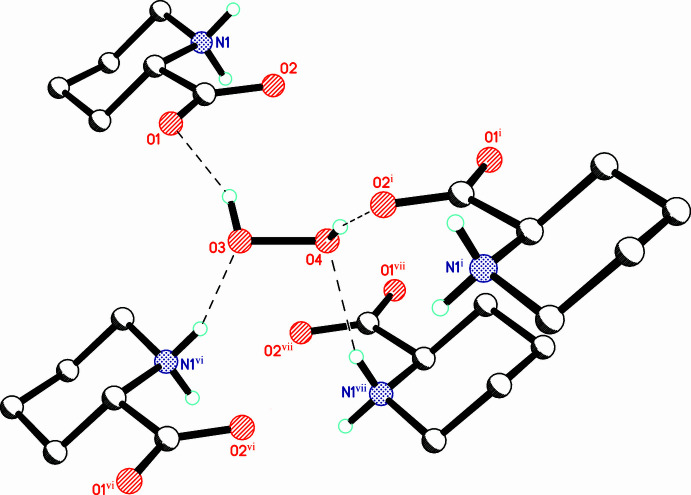
Hydrogen bonds formed by the peroxide mol­ecule H3—O3—O4—H4. Hydrogen bonds are shown as dashed lines. [Symmetry codes: (i) 1 − *x*, 1 − *y*, −*z*; (vi) 1 + *x*, *y*, *z*; (vii) 1 − *x*, 1 − *y*, 1 − *z*.]

**Figure 4:**
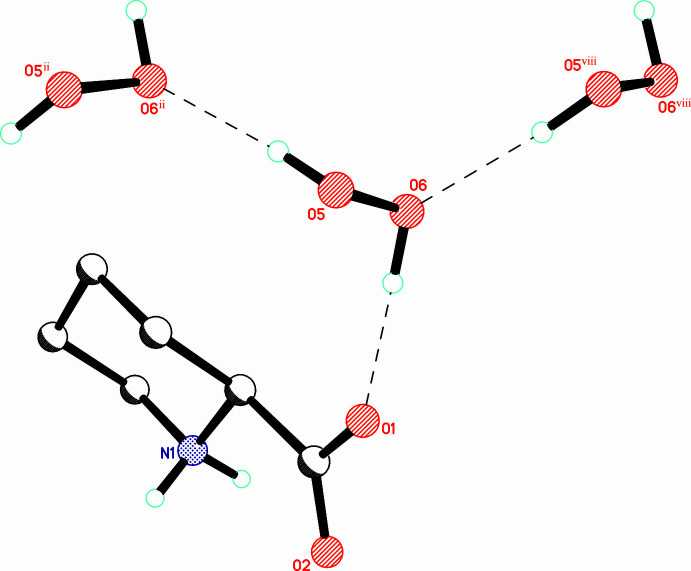
Hydrogen bonds formed by peroxide mol­ecule H5—O5—O6—H6. Hydrogen bonds are shown as dashed lines. [Symmetry codes: (ii) *x*, 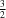 − *y*, 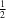 + *z*; (viii) *x*, 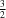 − *y*, −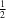 + *z*.]

**Figure 5:**
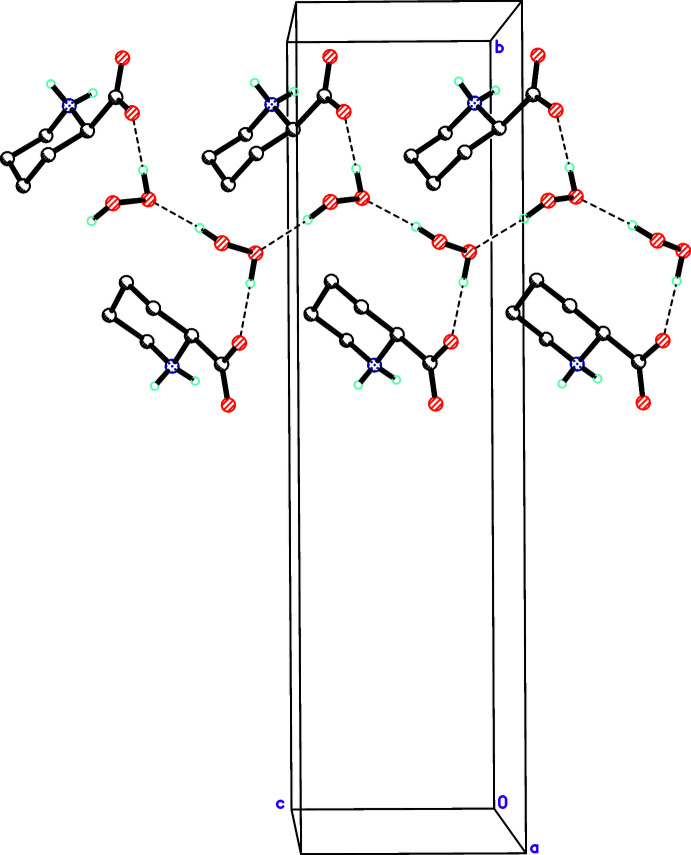
Peroxide hydrogen-bonded chains parallel to the *c* axis. Hydrogen bonds are shown as dashed lines.

In the crystal, the organic mol­ecule acts as a donor of two N^+^—H⋯OHOH, and as an acceptor of three COO^−^⋯HOOH hydrogen bonds (Table 1 ▸, Fig. 2 ▸). The O—O bond lengths [1.4600 (9) and 1.4646 (11) Å] are typical for amino acid peroxosolvates (mean value of 1.465 Å according to the latest, March 2020 version of the CSD; Groom *et al.*, 2016 ▸). Both crystallographically independent peroxide mol­ecules occupy general positions and adopt skew conformations with H—O—O—H torsion angles of 102.5 (15) and −105.1 (15)°. It is well known that peroxide mol­ecules always form at least two donor hydrogen bonds in the structures of organic peroxosolvates (Chernyshov *et al.*, 2017 ▸) and compound (I) is no exception. However, the symmetry-independent peroxide mol­ecules in (I) form a different total number of hydrogen bonds: two donor HOOH⋯^−^O_2_C and two acceptor N^+^—H⋯OHOH for H3—O3—O4—H4 ([2,2] mode; Table 1 ▸, Fig. 3 ▸) and two donor HOOH⋯^−^O_2_C and HOOH⋯OHOH together with one acceptor for H5—O5—O6—H6 ([2,1] mode; Table 1 ▸, Fig. 4 ▸). The occurrence of inter­peroxide hydrogen-bonds results in the formation of simple infinite hydrogen-bonded ‘hydro­peroxo’-linked chains (Grishanov *et al.*, 2017 ▸), running along the *c*-axis direction (Fig. 5 ▸). It is significant that such chains and HOOH⋯OHOH hydrogen bonds were not observed previously in the structures of aliphatic α-amino acid peroxosolvates. The reason for this is that charge-assisted HOOH⋯^−^O_2_C bonds are energetically preferable to HOOH⋯OHOH inter­actions (Jesus & Redinha, 2011 ▸; Zick & Geiger, 2018 ▸). For example, in (I) the only inter­peroxide hydrogen-bond O5—H5⋯O6 is noticeably longer [2.778 (1) Å] than the three HOOH⋯^−^O_2_C bonds [2.641 (1)–2.749 (1) Å].

## Database survey   

Aliphatic α-amino acids contain side chains without heteroatoms suitable for hydrogen-bonding. Up to date, six structures of their peroxosolvates are known: monoperoxosolvates of *N,N*-di­methyl­glycine (C_4_H_9_NO_2_; Kapustin *et al.*, 2014 ▸), *N*-methyl­glycine (sarcosine) (C_3_H_7_NO_2_; Navasardyan *et al.*, 2017 ▸), isoleucine (C_6_H_13_NO_2_; Prikhodchenko *et al.*, 2011 ▸); sesquiperoxosolvates of glycine (C_2_H_5_NO_2_), dl-2-amino­butyric acid (C_4_H_9_NO_2_) and l-phenyl­alanine (C_9_H_11_NO_2_; Prikhodchenko *et al.*, 2011 ▸). In all of these structures, the organic mol­ecules exist as zwitterions and all peroxide hydrogen atoms are involved in charge-assisted hydrogen-bonds with the carboxyl­ate groups. All peroxide mol­ecules adopt skew conformations with H—O—O—H torsion angles varying between 88.6 and 166.3°.

**Figure 6:**
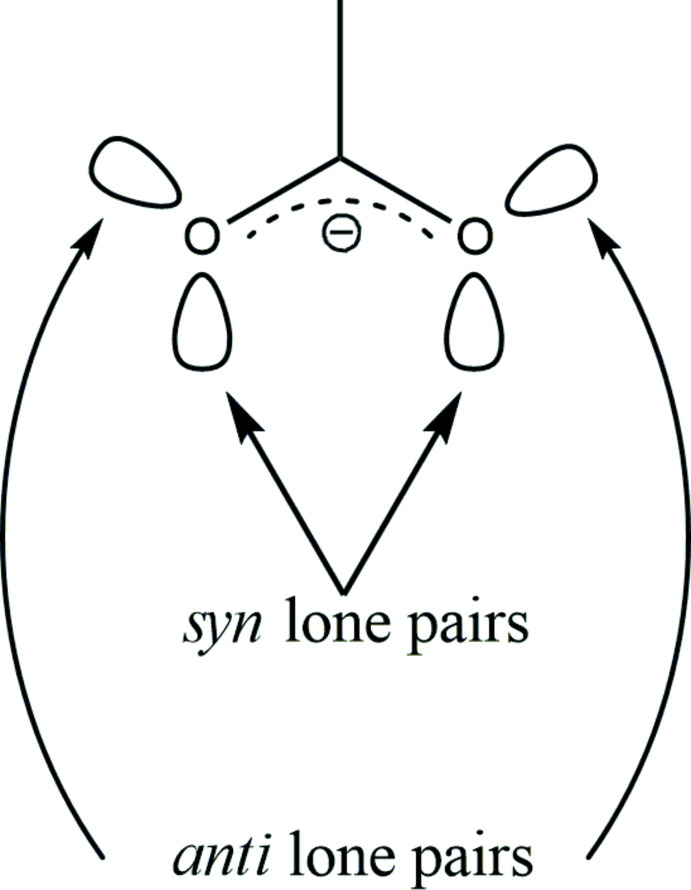
The mutual arrangement of *syn* and *anti* lone electron pairs of the carboxyl­ate anion.

**Figure 7:**
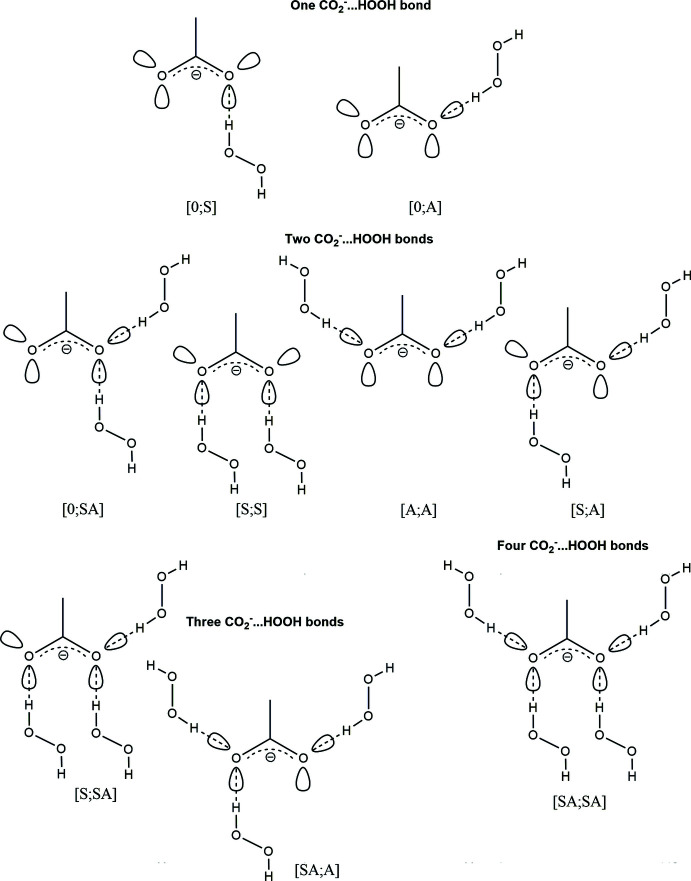
Possible hydrogen-bonded motifs in the structures of amino acid peroxosolvates.

The carboxyl­ate anions possess four *sp* ^2^-hybridized lone electron pairs suitable for hydrogen-bond formation (Fig. 6 ▸) (Mills & Dean, 1996 ▸). It is well known that *syn* and *anti* lone pairs exhibit noticeably different basicity (Gandour, 1981 ▸; Pal *et al.*, 2018 ▸) and hydrogen-bonding properties as a result of electronic and steric effects (Gorbitz & Etter, 1992 ▸; Pranata, 1993 ▸). Nine hydrogen-bonded linkage modes are theoretically possible in the structures of amino acid peroxosolvates, taking into account that bifurcated HOOH⋯O bonds are not known (Fig. 7 ▸). The two simplest modes [0;S] and [0;A] have not been observed in peroxosolvates of α-amino acids, since the peroxide/acid molar ratio is greater than or equal to 1 in each reported structure. The [S;S] linkage was observed in sarcosine monoperoxosolvate. The [S;A] mode was found for the *N,N*-di­methyl­glycine and isoleucine monosolvates. Examples of neither the [0;SA] nor the [A;A] case are currently known. As for three hydrogen bonds, both [S;SA] and [SA;A] linkages were found in the sesquiperoxosolvate structures of glycine, dl-2-amino­butyric acid and l-phenyl­alanine. Following the same logic, we expected to find [SA;SA] in the structure of the title diperoxosolvate (I). However, the triple hydrogen-bonded case [S;SA] occurred, with the fourth donor hydrogen bond HOOH⋯O engaged in forming hydrogen-bonded peroxide chains. It has been shown that the ability of carb­oxy­lic *anti*-orbitals to form hydrogen bonds is strongly affected by steric hindrance caused by β-substituents in the side chains of carb­oxy­lic acids (Gorbitz & Etter, 1992 ▸). It is clear that in (I) the unfeasibility of the fourth carb­oxy­lic hydrogen bond is the result of steric effects caused by the peroxide mol­ecules hydrogen bonded with the ammonium group (Fig. 2 ▸). It should be noted that the spatial arrangement of the endocyclic amino group in (I) is predefined by the aforementioned planarity of the N—C—CO_2_ amino acid fragment.

## Synthesis and crystallization   

96% Hydrogen peroxide was prepared by an extraction method from serine peroxosolvate (Wolanov *et al.*, 2010 ▸). Colourless prismatic crystals of the title compound were obtained by cooling a saturated solution (r.t.) of pipecolinic acid (Aldrich) in 96% hydrogen peroxide to 255 K. Handling procedures for concentrated hydrogen peroxide have been described in detail (danger of explosion!) by Schumb *et al.* (1955 ▸).

## Refinement   

Crystal data, data collection and structure refinement details are summarized in Table 2 ▸. All hydrogen atoms were found in difference-Fourier maps and were refined with independent positional and isotropic displacement parameters.

## Supplementary Material

Crystal structure: contains datablock(s) I. DOI: 10.1107/S205698902000972X/fy2147sup1.cif

Structure factors: contains datablock(s) I. DOI: 10.1107/S205698902000972X/fy2147Isup2.hkl

CCDC reference: 2016846

Additional supporting information:  crystallographic information; 3D view; checkCIF report

## Figures and Tables

**Table 1:** Hydrogen-bond geometry (Å, °)

*D*—H⋯*A*	*D*—H	H⋯*A*	*D*⋯*A*	*D*—H⋯*A*
O3—H3⋯O1	0.872 (17)	1.819 (17)	2.6463 (10)	157.8 (15)
O4—H4⋯O2^i^	0.866 (18)	1.889 (18)	2.7490 (10)	172.2 (15)
O6—H6⋯O1	0.881 (16)	1.760 (16)	2.6412 (10)	177.4 (14)
O5—H5⋯O6^ii^	0.883 (17)	1.898 (18)	2.7777 (12)	174.4 (15)
N1—H1⋯O3^iii^	0.912 (15)	1.961 (15)	2.8336 (11)	159.6 (13)
N1—H2⋯O4^iv^	0.875 (15)	2.112 (15)	2.9459 (11)	159.1 (12)

Symmetry codes: (i) 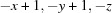; (ii) 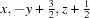; (iii) 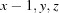; (iv) 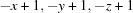.

**Table 2:** Experimental details

Crystal data	
Chemical formula	C_6_H_11_NO_2_·2H_2_O_2_
*M*_r_	197.19
Crystal system, space group	Monoclinic, *P*2_1_/*c*
Temperature (K)	150
*a*, *b*, *c* (Å)	6.5739 (4), 22.9278 (15), 6.0647 (4)
β (°)	93.770 (1)
*V* (Å^3^)	912.12 (10)
*Z*	4
Radiation type	Mo *K*α
μ (mm^−1^)	0.13
Crystal size (mm)	0.50 × 0.50 × 0.50

Data collection	
Diffractometer	Bruker SMART APEXII
Absorption correction	Multi-scan (*SADABS*; Bruker, 2008 ▸)
*T*_min_, *T*_max_	0.659, 0.746
No. of measured, independent and observed [*I* > 2σ(*I*)] reflections	9809, 2418, 2170
*R*_int_	0.018
(sin θ/λ)_max_ (Å^−1^)	0.682

Refinement	
*R*[*F* ^2^ > 2σ(*F* ^2^)], *wR*(*F* ^2^), *S*	0.031, 0.084, 1.08
No. of reflections	2418
No. of parameters	178
H-atom treatment	All H-atom parameters refined
Δρ_max_, Δρ_min_ (e Å^−3^)	0.44, −0.19

Computer programs: *APEX2* and *SAINT* (Bruker, 2008 ▸), *SHELXL2018/3* (Sheldrick, 2015 ▸) and *XP* in *SHELXTL* (Sheldrick, 2008 ▸).

## supplementary crystallographic information

### Crystal data

**Table:**

C_6_H_11_NO_2_·2H_2_O_2_	*F*(000) = 424
*M**_r_* = 197.19	*D*_x_ = 1.436 Mg m^−^^3^
Monoclinic, *P*2_1_/*c*	Mo *K*α radiation, λ = 0.71073 Å
*a* = 6.5739 (4) Å	Cell parameters from 5232 reflections
*b* = 22.9278 (15) Å	θ = 3.1–30.6°
*c* = 6.0647 (4) Å	µ = 0.13 mm^−^^1^
β = 93.770 (1)°	*T* = 150 K
*V* = 912.12 (10) Å^3^	Nugget, colourless
*Z* = 4	0.50 × 0.50 × 0.50 mm

### Data collection

**Table:**

Bruker SMART APEXII diffractometer	2170 reflections with *I* > 2σ(*I*)
ω scans	*R*_int_ = 0.018
Absorption correction: multi-scan (SADABS; Bruker, 2008)	θ_max_ = 29.0°, θ_min_ = 3.1°
*T*_min_ = 0.659, *T*_max_ = 0.746	*h* = −8→8
9809 measured reflections	*k* = −30→31
2418 independent reflections	*l* = −8→8

### Refinement

**Table:**

Refinement on *F*^2^	Primary atom site location: structure-invariant direct methods
Least-squares matrix: full	Secondary atom site location: difference Fourier map
*R*[*F*^2^ > 2σ(*F*^2^)] = 0.031	Hydrogen site location: difference Fourier map
*wR*(*F*^2^) = 0.084	All H-atom parameters refined
*S* = 1.08	*w* = 1/[σ^2^(*F*_o_^2^) + (0.0419*P*)^2^ + 0.218*P*] where *P* = (*F*_o_^2^ + 2*F*_c_^2^)/3
2418 reflections	(Δ/σ)_max_ = 0.001
178 parameters	Δρ_max_ = 0.44 e Å^−^^3^
0 restraints	Δρ_min_ = −0.19 e Å^−^^3^

### Special details

**Table:**

Geometry. All esds (except the esd in the dihedral angle between two l.s. planes) are estimated using the full covariance matrix. The cell esds are taken into account individually in the estimation of esds in distances, angles and torsion angles; correlations between esds in cell parameters are only used when they are defined by crystal symmetry. An approximate (isotropic) treatment of cell esds is used for estimating esds involving l.s. planes.

### Fractional atomic coordinates and isotropic or equivalent isotropic displacement parameters (Å^2^)

**Table:**

	*x*	*y*	*z*	*U*_iso_*/*U*_eq_	
N1	−0.02450 (12)	0.57964 (3)	0.57611 (13)	0.01528 (16)	
H1	−0.093 (2)	0.5604 (6)	0.463 (2)	0.029 (3)*	
H2	0.039 (2)	0.5538 (6)	0.661 (2)	0.025 (3)*	
O1	0.40875 (11)	0.60608 (3)	0.25095 (12)	0.02266 (17)	
O2	0.21298 (11)	0.52860 (3)	0.30218 (11)	0.02005 (16)	
C1	−0.17311 (14)	0.61336 (4)	0.70359 (15)	0.01777 (18)	
H11	−0.2642 (19)	0.5851 (6)	0.759 (2)	0.021 (3)*	
H12	−0.2449 (19)	0.6384 (6)	0.597 (2)	0.024 (3)*	
C2	−0.05768 (15)	0.64708 (4)	0.88702 (15)	0.01993 (19)	
H21	−0.157 (2)	0.6679 (6)	0.968 (2)	0.029 (3)*	
H22	0.010 (2)	0.6192 (6)	0.988 (2)	0.029 (3)*	
C3	0.10082 (16)	0.68727 (4)	0.79508 (17)	0.0218 (2)	
H32	0.033 (2)	0.7172 (6)	0.703 (2)	0.027 (3)*	
H31	0.179 (2)	0.7082 (6)	0.916 (2)	0.030 (3)*	
C4	0.24769 (14)	0.65223 (4)	0.66075 (16)	0.01923 (19)	
H41	0.343 (2)	0.6784 (6)	0.590 (2)	0.027 (3)*	
H42	0.326 (2)	0.6256 (6)	0.759 (2)	0.027 (3)*	
C5	0.13006 (13)	0.61800 (4)	0.47837 (14)	0.01483 (17)	
H52	0.0520 (18)	0.6451 (5)	0.377 (2)	0.019 (3)*	
C6	0.26188 (13)	0.58042 (4)	0.33422 (14)	0.01570 (18)	
O3	0.74598 (11)	0.54761 (3)	0.18291 (11)	0.01986 (16)	
O4	0.72361 (11)	0.48529 (3)	0.13828 (12)	0.02162 (16)	
H4	0.737 (2)	0.4839 (7)	−0.003 (3)	0.040 (4)*	
H3	0.620 (3)	0.5583 (7)	0.194 (3)	0.040 (4)*	
O5	0.67290 (12)	0.72538 (4)	0.33260 (14)	0.02787 (18)	
O6	0.49801 (12)	0.71623 (3)	0.17405 (13)	0.02507 (17)	
H5	0.613 (2)	0.7454 (7)	0.434 (3)	0.041 (4)*	
H6	0.472 (2)	0.6792 (7)	0.199 (2)	0.034 (4)*	

### Atomic displacement parameters (Å^2^)

**Table:**

	*U*^11^	*U*^22^	*U*^33^	*U*^12^	*U*^13^	*U*^23^
N1	0.0163 (3)	0.0143 (4)	0.0153 (3)	−0.0005 (3)	0.0025 (3)	−0.0015 (3)
O1	0.0229 (4)	0.0170 (3)	0.0293 (4)	−0.0015 (3)	0.0110 (3)	−0.0023 (3)
O2	0.0237 (3)	0.0149 (3)	0.0221 (3)	−0.0016 (2)	0.0060 (3)	−0.0044 (2)
C1	0.0169 (4)	0.0183 (4)	0.0185 (4)	0.0023 (3)	0.0044 (3)	−0.0002 (3)
C2	0.0237 (5)	0.0202 (4)	0.0163 (4)	0.0037 (4)	0.0042 (3)	−0.0023 (3)
C3	0.0251 (5)	0.0179 (4)	0.0225 (5)	−0.0009 (4)	0.0036 (4)	−0.0074 (4)
C4	0.0186 (4)	0.0182 (4)	0.0211 (4)	−0.0024 (3)	0.0023 (3)	−0.0057 (3)
C5	0.0165 (4)	0.0132 (4)	0.0150 (4)	−0.0002 (3)	0.0028 (3)	−0.0004 (3)
C6	0.0170 (4)	0.0162 (4)	0.0140 (4)	0.0019 (3)	0.0018 (3)	−0.0012 (3)
O3	0.0193 (3)	0.0172 (3)	0.0229 (3)	0.0006 (3)	0.0008 (3)	−0.0044 (2)
O4	0.0301 (4)	0.0158 (3)	0.0187 (3)	0.0029 (3)	0.0003 (3)	−0.0012 (2)
O5	0.0261 (4)	0.0308 (4)	0.0265 (4)	−0.0044 (3)	0.0001 (3)	−0.0053 (3)
O6	0.0308 (4)	0.0200 (4)	0.0237 (4)	−0.0027 (3)	−0.0029 (3)	0.0024 (3)

### Geometric parameters (Å, º)

**Table:**

N1—C5	1.4955 (11)	C3—H32	0.974 (14)
N1—C1	1.4996 (11)	C3—H31	0.991 (14)
N1—H1	0.912 (15)	C4—C5	1.5247 (12)
N1—H2	0.875 (15)	C4—H41	0.986 (14)
O1—C6	1.2639 (11)	C4—H42	0.975 (14)
O2—C6	1.2429 (11)	C5—C6	1.5353 (12)
C1—C2	1.5166 (13)	C5—H52	0.991 (12)
C1—H11	0.958 (13)	O3—O4	1.4600 (9)
C1—H12	0.966 (13)	O3—H3	0.872 (17)
C2—C3	1.5239 (14)	O4—H4	0.866 (18)
C2—H21	0.968 (14)	O5—O6	1.4646 (11)
C2—H22	0.972 (14)	O5—H5	0.883 (17)
C3—C4	1.5313 (13)	O6—H6	0.881 (16)
			
C5—N1—C1	112.57 (7)	C4—C3—H31	109.3 (8)
C5—N1—H1	107.6 (9)	H32—C3—H31	106.3 (11)
C1—N1—H1	109.2 (9)	C5—C4—C3	110.40 (8)
C5—N1—H2	108.7 (9)	C5—C4—H41	107.7 (8)
C1—N1—H2	110.4 (9)	C3—C4—H41	110.7 (8)
H1—N1—H2	108.2 (12)	C5—C4—H42	110.0 (8)
N1—C1—C2	109.24 (7)	C3—C4—H42	109.4 (8)
N1—C1—H11	106.0 (8)	H41—C4—H42	108.6 (11)
C2—C1—H11	112.4 (8)	N1—C5—C4	109.91 (7)
N1—C1—H12	105.4 (8)	N1—C5—C6	108.62 (7)
C2—C1—H12	112.9 (8)	C4—C5—C6	115.06 (7)
H11—C1—H12	110.4 (11)	N1—C5—H52	106.0 (7)
C1—C2—C3	111.17 (8)	C4—C5—H52	110.1 (7)
C1—C2—H21	107.6 (8)	C6—C5—H52	106.7 (7)
C3—C2—H21	112.8 (8)	O2—C6—O1	125.38 (8)
C1—C2—H22	108.3 (8)	O2—C6—C5	118.40 (8)
C3—C2—H22	109.6 (8)	O1—C6—C5	116.16 (8)
H21—C2—H22	107.3 (11)	O4—O3—H3	101.8 (11)
C2—C3—C4	110.34 (8)	O3—O4—H4	101.7 (10)
C2—C3—H32	109.7 (8)	O6—O5—H5	99.6 (11)
C4—C3—H32	110.2 (8)	O5—O6—H6	100.4 (10)
C2—C3—H31	111.0 (8)		
			
C5—N1—C1—C2	−58.48 (10)	C3—C4—C5—C6	−179.22 (8)
N1—C1—C2—C3	56.95 (10)	N1—C5—C6—O2	6.85 (11)
C1—C2—C3—C4	−56.65 (11)	C4—C5—C6—O2	130.51 (9)
C2—C3—C4—C5	55.85 (11)	N1—C5—C6—O1	−175.64 (8)
C1—N1—C5—C4	58.51 (10)	C4—C5—C6—O1	−51.98 (11)
C1—N1—C5—C6	−174.80 (7)	H3—O3—O4—H4	102.5 (15)
C3—C4—C5—N1	−56.25 (10)	H5—O5—O6—H6	−105.1 (15)

### Hydrogen-bond geometry (Å, º)

**Table:**

*D*—H···*A*	*D*—H	H···*A*	*D*···*A*	*D*—H···*A*
O3—H3···O1	0.872 (17)	1.819 (17)	2.6463 (10)	157.8 (15)
O4—H4···O2^i^	0.866 (18)	1.889 (18)	2.7490 (10)	172.2 (15)
O6—H6···O1	0.881 (16)	1.760 (16)	2.6412 (10)	177.4 (14)
O5—H5···O6^ii^	0.883 (17)	1.898 (18)	2.7777 (12)	174.4 (15)
N1—H1···O3^iii^	0.912 (15)	1.961 (15)	2.8336 (11)	159.6 (13)
N1—H2···O4^iv^	0.875 (15)	2.112 (15)	2.9459 (11)	159.1 (12)

Symmetry codes: (i) −*x*+1, −*y*+1, −*z*; (ii) *x*, −*y*+3/2, *z*+1/2; (iii) *x*−1, *y*, *z*; (iv) −*x*+1, −*y*+1, −*z*+1.
